# Regulation of serum matrix metalloproteinases and tissue inhibitor of metalloproteinases-1 following rituximab therapy in patients with rheumatoid arthritis refractory to anti-tumor necrosis factor blockers

**DOI:** 10.1007/s00296-014-3112-1

**Published:** 2014-09-05

**Authors:** Piotr Adrian Klimiuk, Izabela Domysławska, Stanisław Sierakowski, Justyna Chwiećko

**Affiliations:** Department of Rheumatology and Internal Diseases, Medical University of Bialystok, M.C. Skłodowskiej 24a, 15-276 Białystok, Poland

**Keywords:** MMP-1, MMP-3, MMP-9, TIMP-1, Rheumatoid arthritis, Rituximab

## Abstract

In our article, we evaluated the regulatory effects of the infusions of rituximab, a monoclonal antibody directed against CD20^+^ B cells, on the serum matrix metalloproteinases (MMPs) and tissue inhibitor of metalloproteinases-1 (TIMP-1) levels in patients with active rheumatoid arthritis (RA) not responding to anti-tumor necrosis factor (anti-TNF) therapy. Twelve RA patients were planned to receive four infusions of 1,000 mg of rituximab at weeks 0, 2, 24 and 26. The therapy was combined with methotrexate (MTX) (20–30 mg/week). Seven patients were refractory to previously received infliximab, and five to etanercept. Serum concentrations of interstitial collagenase (MMP-1), stromelysin-1 (MMP-3), gelatinase B (MMP-9) and TIMP-1 were measured by ELISA on weeks 0, 2, 12, 24, 36 and 52. Initial infusion of rituximab downregulated serum MMP-1 (*p* < 0.01), MMP-3 (*p* < 0.001), MMP-9 (*p* < 0.001) and TIMP-1 (*p* < 0.05) levels. Second drug administration caused even more remarkable reduction of measured MMPs (*p* < 0.001 in all cases) and TIMP-1 level (*p* < 0.01). These findings were accompanied by significantly decreased ratios of measured MMPs to TIMP-1. Next rituximab infusions on weeks 24 and 26 sustained the suppression of serum MMPs levels. Prior to the initial rituximab infusion, serum concentrations of studied MMPs and TIMP-1 significantly correlated with markers of RA activity such as disease activity score (DAS28) and CRP levels. Rituximab therapy, beside a rapid clinical improvement, reduced serum MMPs concentrations in RA patients refractory to anti-TNF treatment. Repeated infusions of rituximab maintained initial serum MMPs suppression.

**Electronic supplementary material:**

The online version of this article (doi:10.1007/s00296-014-3112-1) contains supplementary material, which is available to authorized users.

## Introduction

Rheumatoid arthritis (RA) is a chronic, progressive autoimmune disease from which suffer about 1 % of adult population [1]. Morphologically disease is characterized by an invasive and tissue destructive infiltrate of lymphocytes, macrophages and synoviocytes in the joint [2]. Matrix metalloproteinases (MMPs) produced by macrophages and synovial fibroblasts were shown to be involved in the articular tissues destruction [3–5]. The activity of MMPs is downregulated by tissue inhibitor of metalloproteinases (TIMPs), which neutralize MMPs. Therefore, MMPs and TIMPs and especially their imbalance are supposed to be engaged in the process of the joint tissues remodeling in RA [6–8].

Because production of MMPs is controlled among others by TNF-α [5], it was suggested to use anti-TNF agents in the treatment of RA. TNF inhibitors revealed to be very effective at improving signs and symptoms of disease, however, not in all patients [9]. Therefore, a monoclonal antibody directed against CD20^+^ B cells was developed to induce transient depletion of B cells which were shown to stimulate MMPs synthesis by macrophages and fibroblasts [5, 10, 11]. Treatment with rituximab, a monoclonal antibody directed against CD20^+^ B cells, showed significant improvements in disease activity in patients with active RA who had an inadequate response or intolerance to TNF inhibitor [9]. It was even shown that rituximab can inhibit the progression of structural joint damage in these patients [12].

In our previous study, we demonstrated that repeated infusions of the chimeric anti-TNF-α monoclonal antibody (infliximab), beside the decrease in RA activity, reduce serum levels of MMP-1, MMP-3 and MMP-9 [13]. The aim of the present study was to evaluate the effects of the administration of rituximab on serum MMPs and TIMP-1 levels and ratios of MMPs to TIMPs in patients with active RA not responding to anti-tumor necrosis factor therapy.

## Materials and methods

### Patients and samples

A total of 12 patients who fulfilled the American College of Rheumatology 1987 revised criteria for RA [14] were enrolled into an open (non-placebo-controlled) study. None of the patients had previous history of tuberculosis or symptoms of infectious diseases in the last 3 months. Chest X-rays performed before first rituximab infusion were normal in all patients.

Patients recruited to the study had active RA and had an inadequate response to 6 months anti-tumor necrosis factor therapies with infliximab (seven patients) and etanercept (five patients). Clinical and demographic characterization of patients is shown in Table 1. All participants were receiving methotrexate (MTX) (median 25.0 mg/week, range 20–30 mg/week) in a stable dose for at least 3 months and nonsteroidal anti-inflammatory drugs (NSAIDs) in a stable dose for at least 4 weeks before enrollment into the study. Ten patients were receiving corticosteroids (median 10 mg/day of prednisone, range 5–15 mg/day) in a stable dose for at least 4 weeks before beginning of the trial. Such treatment regimen with MTX, NSAIDs and prednison was continued through the whole study.

**Table 1:** Clinical and demographic characterization of RA patients

	RA patients
Sex (F/M)	8/4
Mean age (years)	56.7 ± 11.7
Mean disease duration (years)	14.5 ± 9.4
No. of RF-positive patients	10
No. of patients with anti-citrullinated cyclic peptide antibodies	12
Radiological stage II^a^, patients	2
Radiological stage III^a^, patients	5
Radiological stage IV^a^, patients	5

Data are presented as mean ± SD

*RA* rheumatoid arthritis, *RF* rheumatoid factor

^a^According to Steinbrocker’s criteria

Patients were supposed to have four infusions of 1,000 mg of rituximab at weeks 0, 2, 24 and 26. Blood samples obtained prior infusions from on weeks 0, 2, 24 and additionally on weeks 12, 36 and 52 were clotted for 30 min and next centrifuged for 10 min at 1,000*g*. Serum aliquots were stored at −80 °C. The study protocol was approved by the local ethical committee, and patients’ written consent was obtained.

### Clinical and laboratory assessment

The evaluation included the number of tender joints (of 28 joints assessed), the number of swollen joints (of 28 assessed), erythrocyte sedimentation rate (ESR), patient’s global assessment of disease activity (VAS), disease activity score with four variables (DAS28), C-reactive protein (CRP) concentration measured by radial immunodiffusion kit (Nanorid, The Binding Site Ltd., Birmingham, UK), rheumatoid factor level and anti-citrullinated cyclic peptide (anti-CCP) antibodies measured by commercial ELISA kits (Euroimmun, Medizinische Labordiagnostika AG, Lübeck, Germany). Radiological analysis of the joint destruction was performed according to Steinbrocker’s criteria [15].

### Enzyme-linked immunosorbent assays (ELISA)

The measurements of serum concentrations of interstitial collagenase (MMP-1, matrix metalloproteinases 1), stromelysin-1 (MMP-3), gelatinase B (MMP-9) and tissue inhibitor of metalloproteinases 1 (TIMP-1) were based on a commercial ELISA kits (Biotrak, Amersham Pharmacia Biotech Limited, Little Chalfont Buckinghamshire, England) and performed strictly according to the manufacturer’s instructions.

### Statistical analysis

The normally distributed data were compared by paired Student *t* test. Wilcoxon signed rank test was used to evaluate the differences between non-normally distributed data. Correlations between variables were assessed by Spearman rank order test. *p* values lower than 0.05 were considered statistically significant.

## Results

### Serum levels of MMPs and TIMP-1

**Fig. 1:**
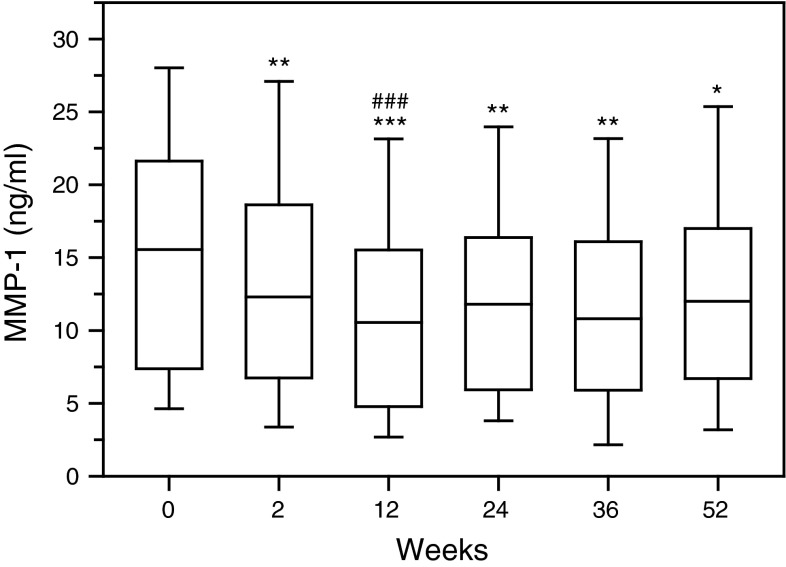
Serum concentrations of interstitial collagenase (MMP-1, matrix metalloproteinase 1) in RA patients, assessed by ELISA technique. Patients were treated with rituximab (1,000 mg) on weeks 0, 2, 24 and 26. Blood samples were obtained on weeks 0, 2, 24 prior to infusion of rituximab, and on weeks 12, 36 and 52. *Box plots* represent median (*line*), 25th and 75th percentiles (*box*), and 10th and 90th percentiles (*whiskers*). Significance of differences between pre-infusion MMP-1 values on week 0 and following weeks was expressed as: *p < 0.05, **p < 0.01, ***p < 0.001. Significance of differences between pre-infusion MMP-1 values on week 2 and following weeks was expressed as: ^#^ *p* < 0.05, ^##^ *p* < 0.01, ^###^ *p* < 0.001

**Fig. 2:**
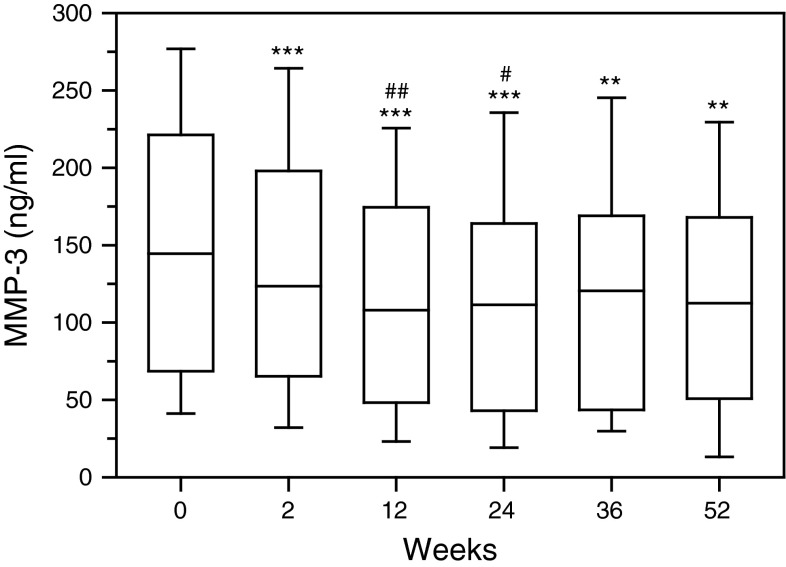
Serum concentrations of stromelysin-1 (MMP-3) in RA patients, assessed by ELISA technique. Patients were treated with rituximab (1,000 mg) on weeks 0, 2, 24 and 26. Blood samples were obtained on weeks 0, 2, 24 prior to infusion of rituximab, and on weeks 12, 36 and 52. *Box plots* represent median (*line*), 25th and 75th percentiles (*box*), and 10th and 90th percentiles (*whiskers*). Significance of differences between pre-infusion MMP-1 values on week 0 and following weeks was expressed as: *p < 0.05, **p < 0.01, ***p < 0.001. Significance of differences between pre-infusion MMP-1 values on week 2 and following weeks was expressed as: ^#^ *p* < 0.05, ^##^ *p* < 0.01

**Fig. 3:**
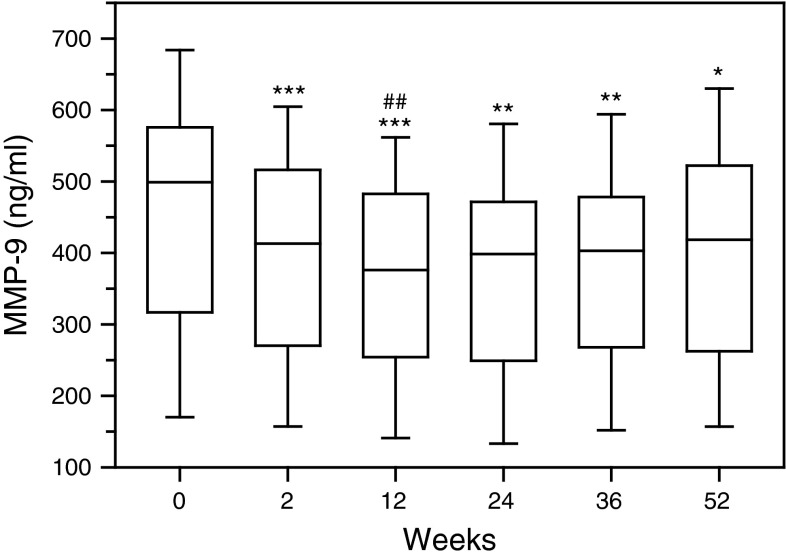
Serum concentrations of gelatinase B (MMP-9) in RA patients, assessed by ELISA technique. Patients were treated with rituximab (1,000 mg) on weeks 0, 2, 24 and 26. Blood samples were obtained on weeks 0, 2, 24 prior to infusion of rituximab, and on weeks 12, 36 and 52. *Box plots* represent median (*line*), 25th and 75th percentiles (*box*), and 10th and 90th percentiles (*whiskers*). Significance of differences between pre-infusion MMP-1 values on week 0 and following weeks was expressed as: *p < 0.05, **p < 0.01, ***p < 0.001. Significance of differences between pre-infusion MMP-1 values on week 2 and following weeks was expressed as: ^#^ *p* < 0.05, ^##^ *p* < 0.01

First rituximab administration caused significant reduction in the concentration of interstitial collagenase (MMP-1) in serum of RA patients (*p* < 0.01) as evaluated on week 2, prior to the next drug infusion (Fig. 1, Figure 1S). The most decreased serum level of MMP-1 was noticed following the second rituximab infusion (*p* < 0.001). Next two administrations of the drug sustained MMP-1 suppression, although were less effective as compared to initial doses of rituximab. Also serum concentration of stromelysin-1 (MMP-3) diminished after rituximab infusion (*p* < 0.001) (Fig. 2, Figure 2S). MMP-3 especially dropped following second rituximab infusion (*p* < 0.001). Further two doses of rituximab maintained MMP-3 suppression in serum of RA patients. Moreover, also serum concentration of gelatinase B (MMP-9) was downregulated in RA patients after rituximab administration (*p* < 0.001) (Fig. 3, Figure 3S). Serum MMP-9 concentration, similar to MMP-1 and MMP-3, decreased especially following second rituximab infusion (*p* < 0.001). Further two drug doses sustained MMP-9 suppression, although were less effective.

**Fig. 4:**
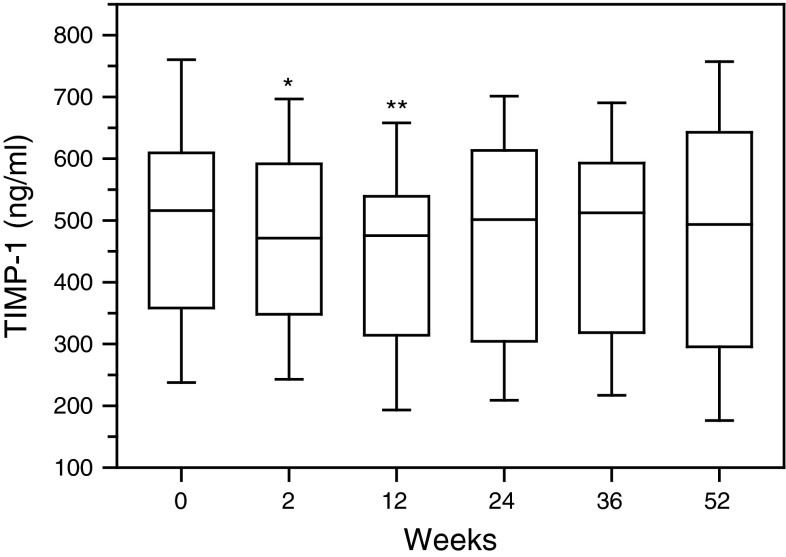
Serum concentrations of tissue inhibitor of metalloproteinases-1 (TIMP-1) in RA patients, assessed by ELISA technique. Patients were treated with rituximab (1,000 mg) on weeks 0, 2, 24 and 26. Blood samples were obtained on weeks 0, 2, 24 prior to infusion of rituximab, and on weeks 12, 36 and 52. *Box plots* represent median (*line*), 25th and 75th percentiles (*box*), and 10th and 90th percentiles (*whiskers*). Significance of differences between pre-infusion MMP-1 values on week 0 and following weeks was expressed as: *p < 0.05, **p < 0.01

Serum levels of TIMP-1 also diminished following first two administrations of rituximab (*p* < 0.05 and *p* < 0.01 respectively) (Fig. 4, Figure 4S). Further observation of serum TIMP-1 revealed even the tendency of their increase toward the initial values.

**Fig. 5:**
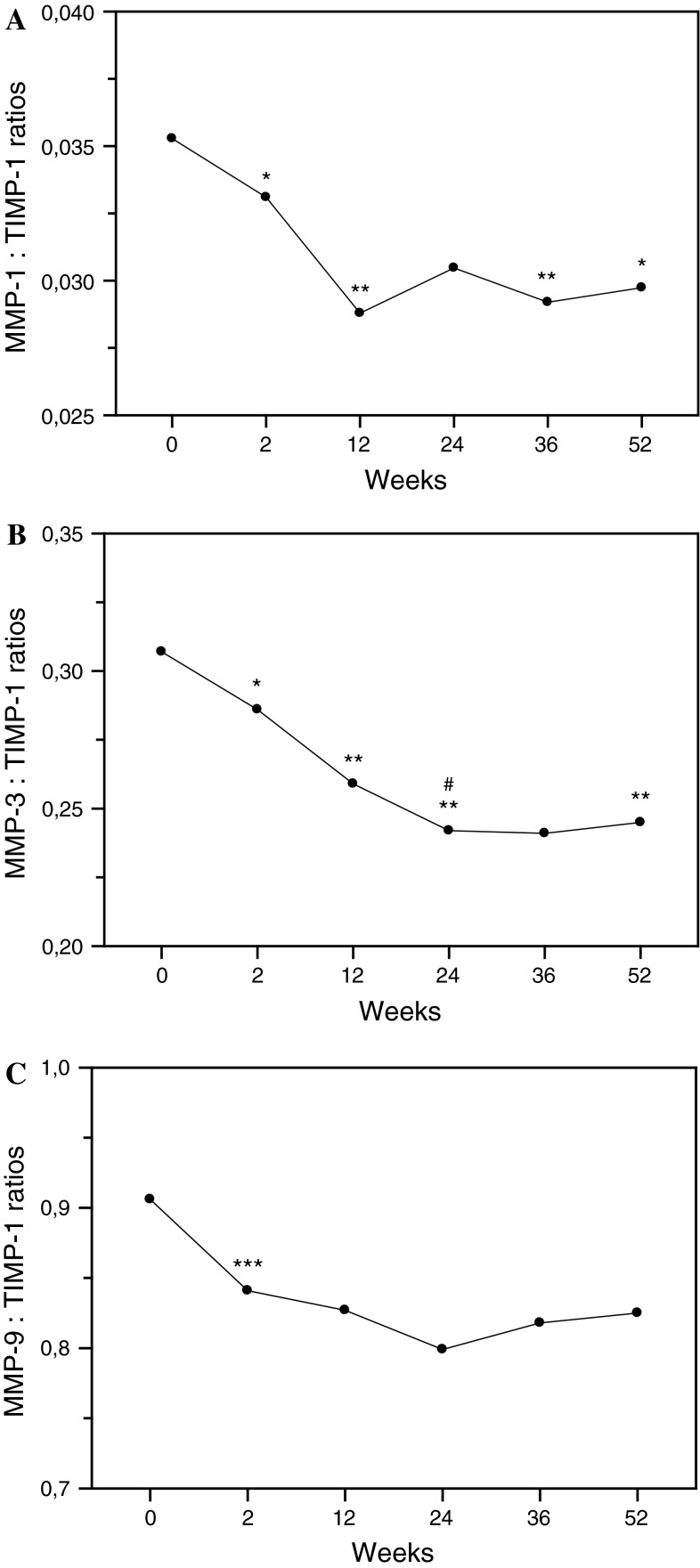
Serum concentration ratios of MMPs to tissue inhibitor of metalloproteinases 1 (TIMP-1). MMP-1:TIMP-1 ratios (**a**). MMP-3:TIMP-1 ratios (**b**). MMP-9:TIMP-1 ratios (**c**). Serum concentrations of metalloproteinases and TIMP-1 in RA patients were assessed by ELISA technique. Patients were treated with rituximab (1,000 mg) on weeks 0, 2, 24 and 26. Blood samples were obtained on weeks 0, 2, 24 prior to infusion of rituximab, and on weeks 12, 36 and 52. Significance of differences between pre-infusion serum concentration ratios of MMPs to TIMP-1 values on week 0 and following weeks was expressed as: *p < 0.05, **p < 0.01, ***p < 0.001. Significance of differences between pre-infusion serum concentration ratios of MMPs to TIMP-1 values on week 2 and following weeks was expressed as: ^#^ *p* < 0.05

Suppression of serum MMPs and TIMP-1 concentrations was accompanied by significantly diminished ratios of analyzed MMPs to TIMP-1 especially marked after second rituximab administration (Fig. 5). Next two doses of rituximab maintained MMPs/TIMP-1 ratios suppression in serum of RA patients.

### Correlations between serum levels of MMPs or TIMP-1 and clinical data

Suppression of serum MMPs and TIMP-1 concentrations were also accompanied by significantly decreased CRP levels especially marked following second rituximab infusion (Figure 5S).

Prior to the initial rituximab administration, serum levels of MMP-1 correlated with marker of RA activity such as the 4 variables disease activity score (DAS28) (*p* < 0.05). Serum concentration of MMP-3, MMP-9 and TIMP-1 correlated with DAS28 (*p* < 0.01, *p* < 0.001 and *p* < 0.05, respectively) and CRP (*p* < 0.01, *p* < 0.05 and *p* < 0.05, respectively)—Table 2. Such associations were also noted after further drug infusions, but were less or no significant. We did not notice any significant correlations between patient age, disease duration or rheumatoid factor with any serum MMPs or TIMP-1 concentrations.

**Table 2:** Correlations between serum concentrations of studied MMPs or tissue inhibitor of metalloproteinases-1 (TIMP-1) and C-reactive protein (CRP) or disease activity score (DAS28) in RA patients before rituximab therapy

	CRP	MMP-1	MMP-3	MMP-9	TIMP-1
DAS28	0.827***	0.643*	0.741**	0.811***	0.643*
CRP		0.508	0.753**	0.701*	0.651*

Data expressed as *r* values (correlation coefficient) according to Spearman rank correlation

* *p* < 0.05; ** *p* < 0.01; *** *p* < 0.001

### Clinical response

**Fig. 6:**
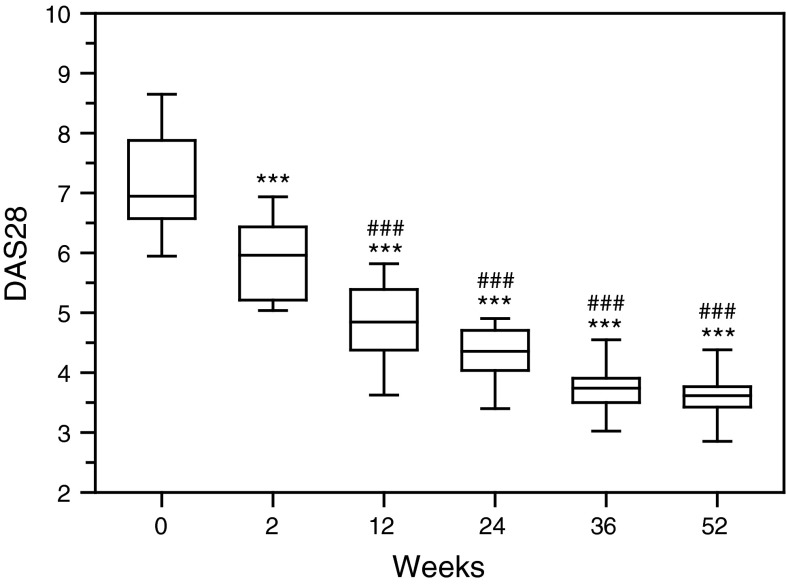
Disease activity score with four variables (DAS28). Patients were treated with rituximab (1,000 mg) on weeks 0, 2, 24 and 26. Blood samples for erythrocyte sedimentation rate (ESR) assessments were obtained on weeks 0, 2, 24 prior to infusion of rituximab, and on weeks 12, 36 and 52. *Box plots* represent median (*line*), 25th and 75th percentiles (*box*), and 10th and 90th percentiles (*whiskers*). Significance of differences between pre-infusion DAS28 values on week 0 and following weeks was expressed as: ***p < 0.001. Significance of differences between pre-infusion DAS28 values on week 2 and following weeks was expressed as: ^###^ *p* < 0.001. Also the differences between pre-infusion DAS28 values on week 12 and following weeks 24, 36 and 52 were significant (*p* < 0.05, *p* < 0.01 and *p* < 0.001, respectively) (data not shown). Furthermore difference between pre-infusion DAS28 value on week 24 and week 52 was significant (*p* < 0.01) (data not shown)

Disease activity score with four variables (DAS28) significantly diminished following initial administration of rituximab (*p* < 0.001). Second drug infusion leaded to even more remarkable drop of DAS28 (*p* < 0.001). Next two doses of the drug caused further decrease of this score (Fig. 6). Reduction of the DAS28 was accompanied by decrease of CRP levels (*p* < 0.001) (data not shown).

## Discussion

Several studies revealed that MMPs and TIMPs, and especially the disturbances of the enzyme to inhibitor ratios, are involved in the degradation of the articular components in the course of RA [3, 6, 8]. Because MMPs production was proven to be under control of such cytokines such as tumor necrosis factor alpha (TNF-α), [5] anti-TNF drugs were suggested for RA therapy. Single [7] and repeated [13] infusions of the chimeric anti-tumor necrosis factor alpha (anti-TNF-α) monoclonal antibody, infliximab, beside the decrease in RA activity, diminished serum levels of MMP-1, MMP-3 and MMP-9. Because further studies revealed that some RA patients may be not responding or intolerant to anti-TNF therapy a monoclonal antibody against CD20^+^ B cells were used to cause transient depletion of B cells which are known to stimulate MMPs production by synovial cells [5, 10, 11]. Therefore, the aim of our present study was to evaluate the effects of the repeated infusions of rituximab, a monoclonal antibody against CD20^+^ B cells, on the serum MMP-1, MMP-3, MMP-9 and TIMP-1 levels, and ratios of measured MMPs to TIMP-1 in patients with active RA refractory to anti-TNF treatment.

MMP-1 called also as interstitial collagenase, produced mainly by synovial fibroblasts and engaged in the destruction of cartilage and synovium [5, 16, 17], was showed to be present in the serum of RA patients [18]. Furthermore, increased MMP-1 levels in early RA [16] correlate with the number of erosions [3], demonstrating its important role in process of joint destruction even in early stages of the disease. In our study, initial rituximab infusion significantly diminished the concentration of MMP-1 in serum of RA patients, which especially dropped after second rituximab infusion. Further two administrations of the drug sustained MMP-1 suppression, although were less effective as compared to initial two doses of rituximab. Subgroup of seven patients pretreated with infliximab shown a stronger reduction of serum levels of MMP-1 following rituximab infusions, compared to the five patients previously treated with etanercept.

Also MMP-3 known as stromelysin-1, whose the main source are fibroblasts, plays an important role in enzyme degradation of several components of extracellular matrix and different types of collagens [17]. Abundance of MMP-3 was observed not only in the serum of long-standing RA patients [18, 19] but also in early stages of the disease [16]. Therefore, MMP-3 was suggested as a useful marker of disease activity in RA [16, 18]. Furthermore, similar MMP-1 also elevated MMP-3 concentrations even in early RA correlate with the number of erosions and disease progression [3, 20]. Thus, it was proposed that MMP-3 may be used in prediction of joint destruction in early RA. We showed that also serum concentration of MMP-3 was downregulated after rituximab administration, especially following second infusion of this drug. MMP-3 suppression in serum of RA patients was maintained by next two doses of this study drug. Decrease of plasma levels of MMP-3 was also shown in a case report of diffuse large B-cell lymphoma, not otherwise specified (DLBCL, NOS) associated with RA treated with six courses of rituximab plus cyclophosphamide, doxorubicin, vincristine and prednisone therapy [21]. However in such complex immunosuppressive therapy courses repeated six times, it is difficult to point which immunosuppressive agent was the most important in demonstrated MMP-3 suppression. Furthermore, diminished serum MMP-3 was also presented in patients with antineutrophil cytoplasmic antibody (ANCA)-associated vasculitis (AAV) treated with rituximab [22].

Gelatinase B (MMP-9), produced mainly by granulocytes, was found in high amounts in sera of RA patients even in early stages of the disease [16]. MMP-9 was shown to be engaged in degradation of not only gelatins but also elastin, aggrecans and link protein [23]. In our study, also serum levels of gelatinase B (MMP-9) decreased in RA patients after rituximab administration. Serum MMP-9 concentration, similar to MMP-1 and MMP-3, diminished especially after second rituximab infusion. Similar to MMP-1 and MMP-3, further two drug doses sustained MMP-9 suppression, although were less effective. It was demonstrated by others that rituximab may decrease serum MMP-9 levels in antineutrophil cytoplasmic antibody (ANCA)-associated vasculitis (AAV) treated with rituximab [24]. Similar to MMP-1, subgroup of 7 patients previously treated with Infliximab had a stronger decrease of serum concentrations of MMP-9 after rituximab infusions, compared to the 5 patients treated with etanercept.

The activity of MMPs is downregulated by TIMPs, their endogenous inhibitors [5]. Furthermore, TIMP-1 was also shown to be a useful marker of fibrosis [25]. Enhanced levels of TIMP-1 were found in RA serum [18] even in early disease [16]. In our study, we revealed that serum TIMP-1 concentration decreases following rituximab administration. However, next drug infusions were followed by the tendency to the increase of serum TIMP-1 levels toward the initial values. Suppressed serum TIMP-1 was also shown in patients with antineutrophil cytoplasmic antibody (ANCA)-associated vasculitis (AAV) treated with rituximab [22].

It was already shown by others that the joint destruction in the pathogenesis of RA may be the result of disturbances between MMPs and TIMPs levels [6, 8]. Recently, we also demonstrated that relative production of TIMPs as compared to MMPs is decreased in RA, and especially in patients with more severe activity of the disease [18] and in early stages of the disease [16]. In the present study, down-regulation of serum MMPs and TIMP-1 levels was accompanied by diminished ratios of measured MMPs to TIMP-1, especially after second rituximab administration.

Observed in our study, only partial suppression of MMPs production caused by rituximab infusions suggests that probably higher doses of this drug and/or more often administrations of these anti-CD20 antibodies are needed in treatment of RA patients refractory to anti-tumor necrosis factor blockers. Other possibility is to use rituximab in combination with other biologic disease-modifying antirheumatic drugs [26]. Because we are aware of the limitation of our study, further research is needed to improve the mode and timing of anti-CD20 antibody therapy in RA.

Previously, we revealed the correlations between MMP-1, MMP-3 and MMP-9 with some markers of disease activity in RA patients [18]. Also in the present study, before the first rituximab administration serum levels of studied MMPs and TIMP-1 correlated with markers of RA activity such as the disease activity score (DAS) or CRP levels. However, after further drug infusions, such associations were less or not significant. Similarly, in case of patients with diffuse large B-cell lymphoma treated with modern chemotherapy with or without CD20 antibody, MMP-9 and TIMP-1 seem to have lost their prognostic value [27].

We are aware that the small number of our patients is a limitation of our study. Furthermore, the correlation between MMPs and rituximab is still fragmentarily known.

In conclusion, monoclonal anti-CD20^+^ B cells antibody therapy (rituximab) combined with MTX, beside a significant clinical improvement, diminished serum MMP-1, MMP-3, MMP-9 and TIMP-1 levels in active RA patients refractory to anti-TNF therapy. These observations were accompanied by the reduced ratios of the measured MMPs to TIMP-1. Further administrations of rituximab sustained MMPs decrease, however, to a lesser extend compared to the first two doses of this drug.

## Electronic supplementary material

Below is the link to the electronic supplementary material.

Supplementary material 1 (DOC 101 kb)

Supplementary material 2 (DOC 102 kb)

Supplementary material 3 (DOC 99 kb)

Supplementary material 4 (DOC 95 kb)

Supplementary material 5 (DOC 65 kb)
