# Supplementary material for: Regulation of serum matrix metalloproteinases and tissue inhibitor of metalloproteinases-1 following rituximab therapy in patients with rheumatoid arthritis refractory to anti-tumor necrosis factor blockers
Source: Rheumatol Int. 2014 Sep 5;35(4):749–55. doi: 10.1007/s00296-014-3112-1 (PMC4365285; doi:10.1007/s00296-014-3112-1)
Supplement: Supplementary file 1 — Supplementary material 1 (DOC 101 kb) [file 296_2014_3112_MOESM1_ESM.doc]

A (Etanercept)

B (Infliximab)

Figure 1 (SUPPLEMENT). Serum concentrations of interstitial collagenase (MMP-1, matrix metalloproteinase 1) in RA patients treated previously with etanercept (5 patients, figure A) and infliximab (7 patients, figure B), assessed by ELISA technique. Patients were treated with rituximab (1000mg) on weeks 0, 2, 24 and 26. Blood samples were obtained on weeks 0, 2, 24 prior to infusion of rituximab, and on weeks 12, 36 and 52. Box plots represent median (line), 25th and 75th percentiles (box), and 10th and 90th percentiles (whiskers). Significance of differences between pre-infusion MMP-1 values on week 0 and following weeks were expressed as: *p <0.05, **p < 0.01. Significance of differences between pre-infusion MMP-1 values on week 2 and following weeks were expressed as: ^#^p <0.05, ^##^p < 0.01.
